# Probiotic Modulation of Gut Microbiota: Antioxidant Mechanisms and Clinical Benefits in Obesity and Type 2 Diabetes Management

**DOI:** 10.3390/antiox15060727

**Published:** 2026-06-08

**Authors:** Hassan Barakat, Hani A. Alfheeaid

**Affiliations:** Department of Food Science and Human Nutrition, College of Agriculture and Food, Qassim University, Buraydah 51452, Saudi Arabia; h.alfheeaid@qu.edu.sa

**Keywords:** gut microbiome, probiotics, functional foods, obesity, type 2 diabetes mellitus, SCFA, next-generation probiotics, food supply

## Abstract

Obesity and type 2 diabetes mellitus (T2DM) represent intertwined global epidemics driven by gut dysbiosis, chronic inflammation, and impaired SCFA production, identifying the microbiome as a therapeutic target. This review synthesizes mechanistic insights and clinical evidence on the role of probiotics as microbiome modulators in the management of metabolic disease. A comprehensive literature search across PubMed, Scopus, Web of Science, and Google Scholar up to May 2026 identified ~230 records using keywords such as probiotics, SCFAs, obesity, and T2DM; a narrative synthesis integrated preclinical, RCT, and meta-analytic data without formal pooling due to heterogeneity. Probiotics restore eubiosis via strain-specific mechanisms, *Lacticaseibacillus rhamnosus* GG enhances tight junctions (ZO-1), *Bifidobacterium breve* BBr60 boosts butyrate cross-feeding, and pasteurized *Akkermansia muciniphila* remodels bile acids (FXR/FGF19), activating G-Protein Coupled Receptor 41 (GPR41)/43-GLP-1 signaling, Treg expansion, and NF-κB suppression. Beyond immunometabolic effects, probiotics mitigate obesity- and T2DM-related oxidative stress by upregulating endogenous antioxidant enzymes (e.g., SOD, catalase, GPx), modulating Nrf2/Keap1 signaling, and reducing lipid peroxidation and other oxidative stress markers in experimental and clinical settings. Meta-analyses of RCTs reveal modest benefits: BMI reductions (~0.3 kg m^−2^), waist circumference (WC) reductions (1–2 cm), HbA1c reductions (0.3–0.4%), and improvements in homeostatic model assessment of insulin resistance (HOMA-IR), especially with multi-strain (>10^9^ CFU day^−1^, ≥12 weeks) synbiotics. Innovative strategies—synbiotics, postbiotics, AI-tailored consortia, and fermented dairy—address engraftment and response variability. Current guidelines recommend 10^9^–10^11^ CFU day^−1^ using multi-strain formulations for 12–24 weeks alongside lifestyle measures, with regimen selection tailored to the dysbiosis phenotype (e.g., NAFLD). Future longitudinal RCTs integrating multi-omics endpoints with AI-driven strain selection should refine—and ultimately individualize—precision probiotic strategies for metabolic therapy.

## 1. Introduction

Obesity and T2DM are interrelated global epidemics caused by accelerated urbanization, dietary changes, sedentary lifestyles, and major disruptions to host metabolism [1]. Beyond these classical risk factors, gut microbiota dysbiosis is an established contributor to the pathogenesis of both disorders [2]. In particular, reduced microbial diversity and loss of beneficial taxa are associated with impaired intestinal barrier function, metabolic endotoxemia, chronic low-grade inflammation, and disrupted energy homeostasis, all of which favor insulin resistance and adiposity [3,4].

In this respect, the gut microbiota actively regulates metabolic health rather than merely marking disease [5]. Dysbiosis affects microbial fermentation, bile acid metabolism, gut hormone signaling, and inflammatory tone, thereby linking intestinal ecology to host glucose and lipid metabolism [6]. Microbial imbalance has been consistently associated with reductions in SCFA-producing bacteria in studies of obesity and T2DM. SCFAs themselves support epithelial integrity, improve insulin sensitivity, and modulate appetite- and glucose-regulating pathways [7]. This mechanistic link positions the microbiome as a promising target for preventive and therapeutic interventions [4,8]. Recent studies on Tongyan Ning further support the microbiota-inflammation-barrier axis, showing that the formula alleviated colitis in mice by reshaping gut microbiota, suppressing inflammatory signaling, and restoring intestinal barrier integrity, while in vitro fermentation studies also demonstrated beneficial changes in fecal microbiota and metabolites [9,10]. Beyond local effects on barrier integrity and inflammation, the gut microbiota also influences extra-intestinal organs through the microbiota–gut–brain axis, a complex bidirectional communication network linking intestinal microbial activity with central neural circuits and metabolic regulation [11]. Recent work in Molecular Neurobiology has further highlighted the microbiota–gut–brain axis as a key interface through which intestinal dysbiosis, immune activation, and microbial metabolites can influence neuroinflammation, synaptic plasticity, and brain function [12].

Therefore, probiotics have attracted attention as microbiome-modulating agents that restore eubiosis and counteract metabolic dysfunction [13]. Probiotics can stimulate SCFA production (especially acetate, propionate, and butyrate) by promoting the growth or metabolic activity of beneficial organisms that exert anti-inflammatory and metabolic benefits in the gut and peripheral tissues [14]. At the same time, probiotics modulate the immune system by improving tight junctions, minimizing intestinal permeability, reducing endotoxin translocation, and altering the immune response to a more regulated, less proinflammatory state [15]. These effects are particularly relevant in obesity and T2DM, where the chronic activation of innate and adaptive inflammatory pathways further exacerbates metabolic impairment [16,17,18].

Clinical evidence supports the potential use of probiotics in the management of metabolic disease [19,20,21]. Meta-analyses and RCTs have reported modest improvements in glycemic control, insulin resistance, inflammatory markers, lipid profiles, and some anthropometric outcomes. However, effect sizes are strain-, dose-, duration-, and host-dependent [20]. Such heterogeneity underscores the need for a more mechanistic and innovation-oriented view of probiotic intervention, encompassing strain selection, delivery technologies, synbiotic and postbiotic formulations, and precision approaches tailored to microbiome profiles and disease stage [22]. These advances suggest that probiotics might be more effective when interventions are tailored to specific microbial and metabolic signatures, rather than using a one-size-fits-all approach [18,23,24].

The present structured literature review, therefore, aims to summarize current knowledge of the mechanisms underlying the link between gut microbiota and obesity/T2DM, assess the clinical impact of probiotic supplementation in these disorders, and discuss emerging strategies to optimize probiotic-based therapies. Finally, the review proposes a translational framework for integrating microbiome-modulating probiotics into broader therapeutic practice, aiming to advance them from adjunctive supplements to evidence-based tools in metabolic disease management. Oxidative stress represents a critical pathophysiological bridge between chronic low-grade inflammation and metabolic dysfunction in obesity and T2DM, contributing to β-cell failure, insulin resistance, and vascular complications. Emerging data indicate that probiotic interventions can modulate redox homeostasis by attenuating ROS production and enhancing endogenous antioxidant defenses, thereby providing a rationale for exploring their antioxidant mechanisms as a therapeutic avenue in these disorders.

## 2. Search Strategy and Methodology

A comprehensive literature search was performed in PubMed, Scopus, Web of Science, and Google Scholar from 2005 to May 2026. Searches combined MeSH and free-text keywords including probiotics, gut microbiota, microbiome, SCFAs, obesity, type 2 diabetes, T2DM, insulin resistance, dysbiosis, eubiosis, immune modulation, and synbiotics, using Boolean operators (AND, OR, NOT) and filters for English-language publications, human studies, and reviews/meta-analyses. We also found other records by searching for reference lists and forward citations.

Eligibility was assessed in two stages: title/abstract screening and full-text review. Inclusion criteria were: (i) original preclinical or clinical studies evaluating probiotics (alone or in synbiotics/postbiotics) in the context of obesity, overweight, metabolic syndrome, insulin resistance, or T2DM; (ii) mechanistic studies describing effects on gut microbiota, metabolic pathways, inflammatory or oxidative stress markers; and (iii) systematic reviews and meta-analyses with a primary focus on probiotic interventions. Exclusion criteria were: (i) studies not involving probiotic or postbiotic interventions (e.g., prebiotics alone, fecal microbiota transplantation without defined probiotic strains); (ii) non-human-only studies without clear translational relevance to obesity or T2DM; (iii) case reports, editorials, letters, conference abstracts without full data; (iv) narrative reviews without original data; and (v) publications lacking metabolic or oxidative stress outcomes of interest or not available in English. Two reviewers independently screened records and resolved disagreements by consensus, yielding approximately >220 primary records after excluding non-relevant designs, non-probiotic interventions, and studies without metabolic or oxidative stress endpoints. Data were extracted on study design, probiotic strains, and dosage, participant characteristics, outcomes, and mechanisms. Narrative synthesis was used to integrate findings across mechanisms, clinical impacts, and innovations. Quantitative data from meta-analyses were summarized qualitatively because of heterogeneity in strains and endpoints.

## 3. Mechanisms of Action

Probiotics—live microorganisms that confer health benefits when administered in adequate amounts—have emerged as adjunctive strategies for managing obesity and T2DM by modulating the gut microbiome, host metabolism, and immune response at multiple levels [25,26]. Experimental and clinical data indicate that probiotic intervention can reshape dysbiotic communities, correct an elevated Firmicutes/Bacteroidetes ratio, enhance SCFA production, and improve gut barrier and inflammatory status, ultimately translating into better glycemic control and reduced adiposity [23,27]. However, these effects are strain-specific and context-dependent, and a mechanistic understanding is needed with a focus on colonization dynamics, SCFA-G protein-coupled receptor (GPR) signaling, bile acid remodeling, immune modulation (especially regulatory T cells, Tregs), and the activities of next-generation strains such as *A. muciniphila* [28,29].

### 3.1. Probiotic Modulation of Oxidative Stress and Antioxidant Defenses

Probiotic strains modulate oxidative stress in obesity and T2DM through several complementary mechanisms at the gut and tissue levels. Experimental models show that *Lactobacillus* and *Bifidobacterium* spp. can reduce systemic and tissue-specific ROS accumulation by enhancing endogenous antioxidant defenses, including increased activities or expression of superoxide dismutase (SOD), catalase (CAT), and glutathione peroxidase (GPx) in the liver, adipose tissue, and pancreas [30,31,32]. These effects are frequently linked to activation of the Nrf2/Keap1 pathway and improved glutathione redox balance, together with reduced lipid peroxidation markers such as malondialdehyde (MDA). In parallel, probiotic-induced reductions in endotoxemia and NF-κB-driven inflammation decrease reactive species generation by immune cells, thereby indirectly attenuating oxidative damage in metabolic tissues [33,34,35]. Clinical studies in obese and T2DM populations have reported that probiotic or synbiotic supplementation can increase total antioxidant capacity, upregulate antioxidant enzyme activities, and reduce circulating markers of oxidative stress (e.g., MDA, oxidized LDL), with these changes often accompanying modest improvements in glycemic control and inflammatory markers [36]. Collectively, these findings support the concept that probiotics exert antioxidant effects both directly, via redox-sensitive signaling and antioxidant enzyme induction, and indirectly, via modulation of inflammation, gut barrier integrity, and SCFA-mediated signaling [37].

### 3.2. Probiotic Colonization and Competitive Exclusion

#### 3.2.1. Establishment and Ecological Integration

Probiotics need to survive gastric acidity and bile after oral intake, adhere to mucus and epithelial surfaces, and successfully compete for nutrients and ecological niches to sustain their effects [38]. Although many conventional probiotics do not permanently engraft, repeated administration can induce lasting shifts in the resident microbial community by altering metabolite profiles and cross-feeding networks [38,39]. Colonization success depends on the baseline microbiota composition, diet (especially fermentable fibers), and host factors such as immune tone and mucus layer integrity, all of which together determine whether exogenous strains are integrated or pass through temporarily [40,41].

#### 3.2.2. Competitive Exclusion of Pathogens

Competitive exclusion, a fundamental ecological process, is how probiotics prevent pathogen colonization by competing for adhesion sites on epithelial cells and mucus, depleting limiting nutrients, and altering local pH and redox conditions [42]. In obesity- and diabetes-associated dysbiosis, this mechanism is particularly relevant for limiting the overgrowth of Gram-negative pathobionts, such as Enterobacteriaceae (*Escherichia coli*, *Enterobacter* spp.), and opportunistic species, such as *Clostridioides difficile* and *Bilophila wadsworthia*, which promote endotoxemia and metabolic inflammation by increasing LPS and other pro-inflammatory metabolites. By promoting beneficial taxa and reducing pathobionts, probiotic supplementation contributes to colonization resistance and attenuates the inflammatory milieu associated with obesity and T2D. A recent meta-analysis of human in vivo trials demonstrated that probiotic supplementation significantly reduced intestinal pathogen colonization (pooled odds ratio ≈ 1.68), consistent with enhanced colonization resistance and improvements in microbial diversity [43]. This colonization resistance is particularly relevant in obesity and diabetes, where dysbiosis often features reduced beneficial taxa and overgrowth of pathobionts that promote endotoxemia and metabolic inflammation [44].

#### 3.2.3. Bacteriocin Production and Reshaping of Firmicutes/Bacteroidetes

Many lactic acid bacteria produce bacteriocins, ribosomally synthesized antimicrobial peptides, that selectively inhibit competing Gram-positive organisms while sparing commensal or beneficial taxa [45]. Bacteriocin-producing probiotic strains can suppress obesogenic Firmicutes and opportunistic pathogens, facilitating the expansion of Bacteroidetes and SCFA-producing commensals, thereby contributing to a normalization of the Firmicutes/Bacteroidetes ratio associated with leanness [46]. Experimental and observational data consistently show that obesity is associated with decreased Bacteroidetes and increased Firmicutes. At the same time, interventions that restore this balance through probiotics, prebiotics, or dietary changes are associated with improvements in adiposity and insulin sensitivity [47].

### 3.3. SCFAs, GPR41/43, and Bile Acids

#### 3.3.1. SCFA Production and Receptor Activation

**Figure 1:**
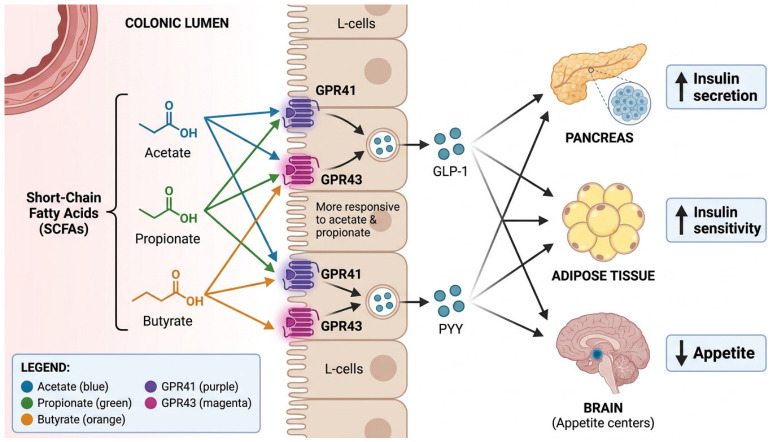
Probiotic-derived SCFAs activate GPR41 and GPR43 on colonic L-cells, triggering GLP-1 and PYY release that promote insulin secretion, improve insulin sensitivity, and suppress appetite in obesity and type 2 diabetes. Figure was created using the FigureLabs AI platform (https://www.figurelabs.ai/).

SCFAs, primarily acetate, propionate, and butyrate, are end products of bacterial fermentation of dietary fibers, and their levels and profiles are strongly influenced by probiotic-induced shifts in microbiota composition [48]. SCFAs signal through two main G protein-coupled receptors on intestinal L cells, adipocytes, and immune cells—GPR41/FFAR3, activated equivalently by propionate and butyrate, and GPR43/FFAR2, more responsive to acetate and propionate [49,50]. GPR43 activation on enteroendocrine L cells stimulates GLP-1 secretion, while GPR41 signaling triggers PYY release, collectively modulating appetite, intestinal transit, and energy homeostasis [51]. Figure 1 shows how probiotic-derived SCFAs, including acetate, propionate, and butyrate, may regulate metabolism by activating GPR41 and GPR43 on colonic L-cells, thereby stimulating GLP-1 and PYY secretion, improving insulin secretion, increasing insulin sensitivity, and reducing appetite in obesity and type 2 diabetes.

#### 3.3.2. GLP-1 Secretion and Glucose Metabolism

Preclinical studies using multistrain formulations such as VSL#3 demonstrate that probiotic-induced increases in butyrate and other SCFAs drive GLP-1 secretion, resulting in improved glucose tolerance, enhanced insulin secretion, and reduced body weight gain in high-fat diet (HFD) and genetic models of obesity and diabetes [52]. Mechanistically, the SCFA-GPR41/43-GLP-1 axis promotes pancreatic β-cell function, improves insulin sensitivity, and reduces hepatic insulin resistance, as shown by genetic models of GPR41/43 deficiency and GLP-1 receptor perturbation [49,53]. In addition, human and animal data suggest that SCFAs stimulate GLP-1 and PYY response, which impact central appetite regulatory circuits and adipocyte biology, providing a mechanistic rationale for probiotic-mediated benefits in weight and glycemic control [54].

#### 3.3.3. Probiotic Modulation of Bile Acid Metabolism

Besides SCFAs, probiotics also influence lipid metabolism by deconjugating and transforming bile acids [55]. Many *Lactobacillus* and *Bifidobacterium* strains express bile salt hydrolases that deconjugate primary bile acids, altering their reabsorption and detergent capacity, and reducing micellar solubilization and intestinal absorption of dietary fat [56]. Probiotics indirectly modulate the gut community and the bile acid pool, affecting the farnesoid X receptor (FXR) and Takeda G-Protein Receptor 5 (TGR5) signaling in the intestine and liver, pathways central to glucose and lipid metabolism [57]. *A. muciniphila*, in particular, has been shown to reprogram bile acid profiles and activate the intestinal FXR–FGF15 axis, reducing hepatic bile acid synthesis, improving glucose tolerance, and limiting hepatic steatosis in HFD-induced metabolic disease models [58].

### 3.4. Immune Modulation, Tregs, and NF-κB

#### 3.4.1. Treg Induction and Anti-Inflammatory Cytokines

Across probiotic studies, a consistent mechanistic theme is enhanced generation of regulatory immune phenotypes, notably CD4^+^CD25^+^FOXP3^+^ Tregs, regulatory B cells, and follicular regulatory T cells [55]. SCFAs derived from probiotic-modulated microbiota directly induce Treg differentiation via FFAR2 (GPR43) signaling and epigenetic effects (histone deacetylase inhibition), whereas microbial polysaccharides and metabolites condition dendritic cells toward a tolerogenic phenotype that further favors Treg generation [59,60]. In models of gestational diabetes and metabolic inflammation, oral probiotic administration increased the frequencies of Treg and Tfr cells. It elevated Interleukin-10 (IL-10) and Transforming Growth Factor Beta (TGF-β), while reducing TNF-α and Interleukin-6 (IL-6), thereby dampening systemic inflammation and improving metabolic parameters [52].

#### 3.4.2. NF-κB Suppression and Metabolic Outcomes

NF-κB is a central transcription factor linking metabolic stress, hyperglycemia, and inflammation; its activation upregulates pro-inflammatory cytokines, chemokines, and adhesion molecules that promote insulin resistance and β-cell dysfunction [55]. Multiple probiotic strains modulate NF-κB signaling at the epithelial and systemic levels, reducing nuclear translocation of p65 and downstream inflammatory gene expression in response to cytokines or endotoxin [61]. By enhancing Treg-mediated suppression and attenuating NF-κB-driven inflammation, probiotics improve insulin sensitivity, adipose tissue inflammation, and overall glucose homeostasis in obesity and type 2 diabetes models [62].

### 3.5. Barrier Integrity and Specific Strains

#### 3.5.1. *L. rhamnosus* GG and Tight Junction Upregulation

*L. rhamnosus* GG (LGG) is one of the most extensively characterized probiotic strains and exerts potent barrier-protective effects [63]. In human enteroid and colonoid models, interferon-γ and TNF-α markedly downregulate tight junction proteins ZO-1 and occludin, increasing paracellular permeability. In contrast, pretreatment with LGG or its conditioned medium normalizes their expression and restores transepithelial electrical resistance [63]. Similar findings in epithelial monolayers show that LGG maintains ZO-1 distribution and TER during cytokine challenge, associated with reduced nuclear translocation of NF-κB p65 and lower CXCL8 expression [56]. In vivo, *L. rhamnosus* strains increase ZO-1 expression and ameliorate barrier dysfunction, thereby reducing endotoxin translocation and metabolic inflammation implicated in obesity-related insulin resistance [64].

Postbiotic components of LGG also contribute to barrier reinforcement. The LGG-derived protein HM0539 enhances mucin production and preserves ZO-1 localization under LPS or TNF-α challenge, with functional improvements in Transepithelial Electrical Resistance (TEER), highlighting that secreted molecules can recapitulate key probiotic effects on the epithelial barrier [65]. Collectively, these data support a model in which LGG upregulates tight junction proteins (including ZO-1) and mucin, stabilizes the mucus-epithelial interface, and attenuates inflammatory signaling, all of which are critical for controlling metabolic endotoxemia in obesity and diabetes [52].

#### 3.5.2. *B. breve* and Butyrate-Linked Effects

*B. breve* is a key member of the infant and adult microbiota and has been increasingly studied for metabolic benefits mediated through SCFA modulation [66]. Clinical data from individuals with obesity indicate that *B. breve* BBr60 supplementation significantly elevates fecal butyrate levels, with parallel improvements in body weight, body fat percentage, and HOMA-IR, and reductions in pro-inflammatory IL-1β alongside increased IL-27 and GLP-1 [66]. Correlation analyses from this trial show that butyrate concentrations positively correlate with GLP-1 and IL-27 and negatively with IL-1β, supporting a mechanistic axis whereby *B. breve* reshapes the microbiota–SCFA–inflammation/hormone network to ameliorate obesity-related metabolic disturbances [55,66].

Although *Bifidobacterium* species are not classical butyrate producers, they engage in cross-feeding interactions by fermenting oligosaccharides to acetate and lactate, which are then converted to butyrate by other commensals (e.g., *Faecalibacterium prausnitzii*), effectively increasing colonic butyrate in a community context [67]. Additional trials in prediabetic and diabetic populations report that *B. breve*-based interventions increase butyrate-producing bacteria and are associated with reductions in HbA1c and fasting plasma glucose, further linking *B. breve* to butyrate-dependent, modest improvements in glycemic control [68,69]. Taken together, these findings are preliminary, and clinical recommendations based on *B. breve* BBr60 remain premature; additional, larger, and independent randomized trials are required before firm conclusions can be drawn about its therapeutic role in obesity or type 2 diabetes.

#### 3.5.3. *A. muciniphila* and Mucin Repair

*A. muciniphila*, a mucin-degrading Verrucomicrobia, is regarded as a next-generation probiotic because its abundance is inversely associated with obesity, insulin resistance, and metabolic syndrome [70,71,72]. By residing in the mucus layer and utilizing mucin as its primary carbon and nitrogen source, *A. muciniphila* stimulates mucin turnover and compensatory synthesis by goblet cells, thereby thickening the mucus barrier, enhancing tight-junction expression, and lowering gut permeability and lipopolysaccharide (LPS) translocation, which collectively reduces systemic endotoxemia, adipose tissue inflammation, and insulin resistance [73,74]. Preclinical studies demonstrate that supplementation with live or pasteurized *A. muciniphila* attenuates body-weight gain, improves glucose tolerance, and alleviates high-fat diet (HFD)-induced fatty liver disease, in part through modulation of bile acid metabolism, short-chain fatty acid (SCFA) production (especially propionate and butyrate), and activation of the intestinal FXR–FGF15/19 axis [74,75].

Consistent with these preclinical data, clinical studies in overweight or obese adults with insulin resistance report improved insulin sensitivity and more favorable lipid profiles following administration of pasteurized *A. muciniphila*, underscoring the translational relevance of its barrier- and bile acid-mediated actions in obesity and type 2 diabetes [74,76]. Recent bibliometric and mechanistic reviews further emphasize the pleiotropic actions of *A. muciniphila* on glucose, lipid, and bile acid metabolism, including promotion of GLP-1 production, reduction in inflammatory mediators, and modulation of the gut–brain axis via neurotransmitter (e.g., Gamma-Aminobutyric Acid (GABA)) production, collectively contributing to improved insulin sensitivity and body-weight regulation [67,74,76,77]. These attributes position *A. muciniphila* as a promising candidate for targeted microbiome-based therapies in obesity and type 2 diabetes [70,74]. Accordingly, *A. muciniphila* should at present be regarded as an investigational next-generation candidate rather than a conventional probiotic. However, the strain-specific mechanistic signatures of key probiotic and next-generation strains have been illustrated in Table 1.

### 3.6. Global Microbiome Modulation and Metabolic Pathways

#### 3.6.1. Community-Level Shifts and Metabolic Signatures

Probiotic interventions affect not only the introduced strains but the broader microbial ecosystem, altering diversity, functional gene content, and metabolite profiles relevant to energy and glucose metabolism [80]. Probiotic supplementation may increase diversity and support the recovery of SCFA-producing taxa and other beneficial functional groups. In contrast, changes in phylum-level proportions, including the Firmicutes/Bacteroidetes ratio, appear more variable across studies [87]. Current microbiome research increasingly emphasizes taxonomic and functional resolution at the genus/strain level and the integration of metagenomic and metabolomic readouts, such as SCFA profiles, bile acid signatures, and LPS-related pathways, as more informative indicators of metabolic risk and probiotic response than phylum-level ratios. These community changes alter the production of SCFAs, bile acids, indoles, and branched-chain amino acids, which regulate host pathways that control insulin sensitivity, adipogenesis, and hepatic lipid accumulation [88]. Interestingly, diabetic patients with hepatocellular carcinoma after hepatitis C virus eradication exhibit a persistent gut microbiome signature characterized by proinflammatory and carcinogenic features. These findings highlight potential targets for microbiome-based risk stratification and therapeutic interventions in this high-risk population [89].

#### 3.6.2. Immune Response and Systemic Inflammation

Probiotics indirectly restore the balance of mucosal and systemic immune response by modulating the microbiome [15]. Increased SCFAs and health-promoting taxa (e.g., Bifidobacterium, Akkermansia) lead to Treg expansion and the production of anti-inflammatory cytokines, while decreasing Th1/Th17 response and pro-inflammatory mediators such as TNF-α, IL-6, and IL-1β [88,90]. This immune reprogramming reduces low-grade metabolic inflammation, the key driver linking obesity and insulin resistance to downstream complications such as type 2 diabetes and cardiovascular disease [91]. Data from clinical and pre-clinical studies suggest that normalization of systemic inflammatory markers induced by probiotics is associated with modest improvements in insulin sensitivity, β-cell function, and glycemic control, supporting the concept of an immunometabolic axis as a key target of probiotic therapy [62].

#### 3.6.3. Strain-Specific Effects and Health Outcomes

Strain specificity is a recurring theme in probiotic research. These barrier-protective and NF-κB-modulating functions of LGG are prominent in enteroid models and are not mimicked by closely related lactobacilli such as *L. crispatus* [63]. *B. breve* strains vary in their ability to elevate butyrate, modulate inflammatory cytokines, and improve clinical end points in obesity and prediabetes, emphasizing the importance of accurate strain characterization [92,93]. *A. muciniphila* is unique as a next-generation mucus-adapted probiotic with strong effects on mucin repair, bile acid metabolism, and systemic metabolic parameters in several models of obesity, fatty liver disease, and type 2 diabetes [74,94].

Probiotics collectively affect obesity and diabetes through colonization and a competitive-exclusion network, SCFA-induced GPR41/43 activation and GLP-1 release, bile acid deconjugation and FXR/TGR5 signaling, immune modulation by Treg cells and NF-κB suppression, and barrier enhancement via upregulation of tight junctions and mucins [92]. Strain-specific effects of *L. rhamnosus* GG, *B. breve*, and *A. muciniphila* exemplify the potential of targeted microbiome interventions to reshape barrier function, SCFAs, bile acid profiles, and immunometabolic pathways, thereby improving weight and glycemic outcomes in experimental and early human studies [56,92].

**Figure 2:**
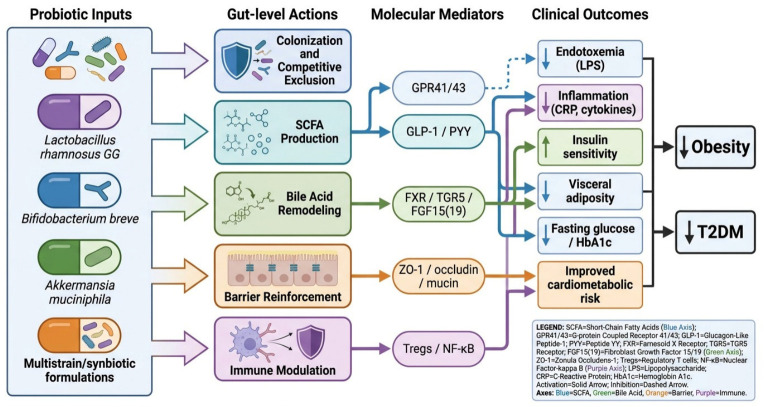
Integrated mechanism map of probiotics in obesity and type 2 diabetes. Probiotic inputs, including *L. rhamnosus* GG, *B. breve*, *A. muciniphila*, and multistrain/synbiotic formulations, act at the gut level through colonization and competitive exclusion, enhanced SCFA production, bile acid remodeling, barrier reinforcement, and immune modulation. These actions converge on molecular mediators such as G protein-coupled receptors GPR41/43, glucagon-like peptide-1 (GLP-1) and peptide YY (PYY), farnesoid X receptor (FXR), Takeda G protein-coupled receptor 5 (TGR5), fibroblast growth factor 15/19 (FGF15/19), tight junction and mucus components (zonula occludens-1, ZO-1; occludin; mucin), and regulatory immune pathways (regulatory T cells, Tregs; nuclear factor kappa-B, NF-κB). Collectively, these pathways reduce endotoxemia [lipopolysaccharide (LPS)] and inflammation [C-reactive protein (CRP), cytokines], improve insulin sensitivity, decrease visceral adiposity, lower fasting glucose and glycated hemoglobin (HbA1c), and ultimately lower cardiometabolic risk, thereby mitigating obesity and type 2 diabetes. Solid arrows denote activation pathways, while dashed arrows indicate inhibitory effects; colors group pathways according to predominant mechanism: blue, SCFA signaling; green, bile acid signaling; orange, barrier function; purple, immune modulation. Figure was created using the FigureLabs AI platform (https://www.figurelabs.ai/).

Probiotics have various functions in obesity and type 2 diabetes. Figure 2 shows the effects of probiotics on the colonization of the gut, the production of SCFAs, the metabolism of bile acids, the integrity of the barrier, and the regulation of the immune system. These actions are mediated through GPR41/43, GLP-1 and PYY, FXR/TGR5/FGF15, tight junction proteins, and Treg/NF-κB signaling pathways, leading to reduced inflammation, improved insulin sensitivity, reduced visceral adiposity, and improved glycemic control. The figure suggests that probiotics may promote metabolic health through multiple complementary mechanisms rather than through a single pathway. Future work should focus on controlled, strain-resolved clinical trials combining metagenomics, metabolomics, and immunophenotyping to refine probiotic strategies for obesity and type 2 diabetes.

## 4. Impact on Obesity

### 4.1. Evidence from Meta-Analyses on Anthropometric Outcomes

Across RCTs in overweight and obese adults, multiple meta-analyses converge on a modest but statistically significant anti-obesity effect of probiotic and synbiotic supplementation, particularly when multistrain formulations are administered at daily doses above 10^9^ CFU for at least 8–12 weeks. Pooled analyses generally show mean BMI reductions of approximately 0.2–0.3 kg/m^2^, with some subgroup analyses in higher-dose or adolescent populations approaching or exceeding 0.5–1.0 kg/m^2^, alongside small but consistent decreases in body weight, fat percentage, and WC versus placebo [95]. A meta-analysis of 15 RCTs (*n* = 957 adults with overweight or obesity) reported reductions in body weight (~0.6 kg), BMI (~0.27 kg/m^2^), and body fat percentage (~0.6%); between-study heterogeneity was substantial, and effect sizes were small at the population level [96].

When probiotics and synbiotics are combined, more recent quantitative syntheses confirm reductions in body weight (≈0.9 kg), BMI (≈0.28 kg/m^2^), and WC (≈1.1 cm), indicating that microbiome-directed “biotics” exert clinically relevant, though modest, improvements in anthropometric indices. One meta-analysis focused on overweight and obese adults found weighted mean differences of roughly −0.55 to −0.60 kg for body weight, −0.29 to −0.30 kg m^2^ for BMI, and nearly −1.0 cm for WC, with subgroup analyses suggesting stronger effects at higher doses and longer intervention durations [95]. In pediatric populations, a Bayesian network meta-analysis in children and adolescents with overweight or obesity showed that multistrain probiotic supplementation significantly reduced BMI (≈−2.1 kg/m^2^) and WC (≈−1.3 cm), further supporting the superior efficacy of multistrain preparations compared with single-strain products in modulating obesity-related traits [97].

Multistrain formulations administered for at least 12 weeks have been reported in several systematic reviews to be more effective in reducing BMI by 0.5–1.0 kg/m^2^ and achieving measurable loss of fat mass, particularly in subjects with marked adiposity or coexisting cardiometabolic risk factors [78,96]. Meta-analyses that specifically examine probiotic and synbiotic interventions in overweight or obese cohorts show improvements in total body fat mass and fat percentage that mirror changes in BMI, suggesting that these interventions alter body composition rather than simply affecting fluid balance [95,96]. Nevertheless, the magnitude of effect remains modest and is highly dependent on strain composition, background diet, initial microbiome configuration, and co-interventions such as caloric restriction or structured physical activity [78,95].

### 4.2. Mechanistic Insights: SCFAs, Histone Deacetylase (HDAC) Inhibition, Fasting-Induced Adipocyte Factor (FIAF), and Energy Harvest

The anti-obesity effects of probiotics are closely related to their ability to reshape the gut microbial ecosystem toward SCFA-generating taxa (e.g., *Bifidobacterium*, *Lactobacillus* spp., and *A. muciniphila*), thereby modulating the production of microbial metabolites that regulate adipose tissue biology and systemic energy homeostasis [80]. SCFAs, primarily acetate, propionate, and butyrate, serve as signaling molecules via free fatty acid receptors FFAR2/3 and as epigenetic modulators by inhibiting HDACs in adipocytes and other metabolic tissues [98]. In vitro and in vivo studies show that butyrate and propionate inhibit HDAC activity in adipose tissue, increasing histone acetylation at promoters of genes involved in lipid oxidation and mitochondrial function, activating pathways such as MKK3/p38/PRAK, and dampening pro-adipogenic signaling, collectively favoring enhanced lipolysis and reduced adiposity [81].

Animal models of diet-induced obesity demonstrate that butyrate administration reduces body weight gain and adiposity while improving glucose homeostasis, in part by hyperacetylating histone H3 and selectively suppressing HDAC activity, independent of FFAR2/3 signaling [81]. These epigenetic effects are complemented by SCFA-mediated upregulation of uncoupling proteins and adiponectin receptor genes in adipose tissue, promoting energy expenditure and more efficient lipid handling [81]. Probiotic-induced enhancement of SCFA production, therefore, constitutes an epigenetic axis through which the microbiota can program adipocyte lipolysis, adipokine secretion, and thermogenic gene expression, providing a mechanistic basis for the modest but consistent reductions in fat mass observed in human trials [80].

FIAF (angiopoietin-like protein 4) represents another key node linking gut microbiota to lipid storage [99]. FIAF inhibits LPL, restraining triglyceride uptake into adipocytes; when dysbiotic microbiota suppress intestinal FIAF, LPL activity increases, triglyceride storage is enhanced, and energy harvest from dietary substrates is augmented [99]. Microbial communities associated with obesity often exhibit reduced FIAF expression and an increased capacity to extract energy from polysaccharides, promoting positive energy balance and adipocyte hypertrophy [98]. In contrast, probiotic interventions that shift the microbiota toward beneficial taxa have been associated in experimental models with normalization of FIAF signaling, partial restoration of SCFA profiles, and attenuation of the high-energy-harvest phenotype, thereby limiting further weight gain and adipose expansion [100].

**Figure 3:**
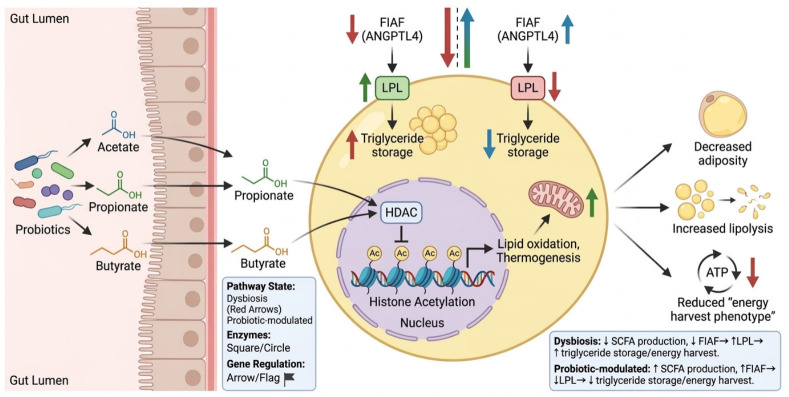
Dysbiosis is shown in red, whereas probiotic-modulated conditions are shown in green/blue. Circles or squares represent enzymes, and gene regulation is indicated by arrows only. SCFAs, FIAF (ANGPTL4), LPL, histone acetylation, lipid oxidation, thermogenesis, and energy harvest are shown as labeled pathway components. Figure was created using the FigureLabs AI platform (https://www.figurelabs.ai/).

Beyond FIAF, probiotics reshape energy harvest by altering microbial gene repertoires encoding carbohydrate-active enzymes, bile salt hydrolases, and nutrient transporters, thereby influencing the efficiency of carbohydrate and lipid digestion and absorption [80]. Human-origin probiotic consortia have been shown to increase fecal SCFA output and shift the Firmicutes/Bacteroidetes ratio, both of which correlate with reduced visceral adipose tissue and improved metabolic indices [80]. In addition, changes in branched-chain and aromatic amino acid metabolism are increasingly recognized as contributors to insulin resistance and type 2 diabetes because elevated circulating amino acid signatures track with impaired metabolic control and altered gut microbial function [101]. Probiotic or diet-based interventions may help attenuate these diabetogenic signatures by shifting the gut ecosystem toward fiber-fermenting taxa that support short-chain fatty acid production and broader metabolic homeostasis [102]. Collectively, these mechanistic data support a model in which probiotic-induced remodeling of the microbiome metabolome—including SCFAs, bile acids, and indoles—attenuates the high-energy-harvest, pro-adipogenic milieu of obesity and instead promotes a phenotype characterized by enhanced lipolysis, reduced lipogenesis, and improved insulin sensitivity [88,103]. Figure 3 illustrates dysbiosis versus probiotic-modulated states.

### 4.3. RCTs: WC and Visceral Fat

In addition to effects on global indices such as weight and BMI, several RCTs have evaluated the impact of probiotic and synbiotic supplementation on central adiposity, particularly WC and visceral adipose tissue [95]. Systematic reviews of biotic interventions in overweight and obese subjects report that multiple trials document significant reductions in WC, typically about 1–2 cm, accompanied by decreases in visceral fat area or sagittal abdominal diameter when multistrain products are administered for 8–12 weeks [95]. Kadooka et al. [104], for example, showed that consumption of milk fermented with *L. gasseri* SBT2055 (10^6^–10^7^ CFU g^−1^) for 12 weeks significantly reduced visceral fat area, BMI, and waist and hip circumferences relative to control fermented milk, with changes evident by week 8 and maintained through week 12.

A double-blind RCT using a multistrain probiotic blend in adults with features of metabolic syndrome demonstrated significant reductions in body fat mass, body weight, BMI, WC, waist-to-height ratio, visceral adipose tissue, and liver steatosis grade compared with placebo, indicating preferential effects on central adiposity and ectopic fat depots [105]. Another study combining *B. breve* and *L. plantarum* in obese adults reported significant decreases in WC and the visceral-to-subcutaneous fat area ratio after 12 weeks, suggesting a beneficial redistribution of fat away from metabolically deleterious visceral stores [106]. Pediatric interventions with multistrain probiotics in obese children and adolescents likewise demonstrate improvements in BMI and WC [107]. However, some of the observed BMI change is attributable to linear growth rather than pure fat mass loss [108].

Synbiotic formulations (probiotic plus prebiotic) frequently yield at least comparable, and in certain trials superior, effects on WC and visceral adiposity relative to probiotics alone [109]. A multispecies synbiotic given for 12 weeks to overweight and obese adults produced a significant 2.57% reduction in WC and decreased body fat percentage, together with improvements in Firmicutes abundance and the Firmicutes/Bacteroidetes ratio [110]. However, not all anthropometric differences versus placebo were statistically significant at the study endpoint [110]. Other synbiotic RCTs in obese women and children confirm significant reductions in BMI z-score, WC, and body fat percentage, with improvements in central adiposity often confined to the synbiotic arm, implying that prebiotic substrates potentiate the ability of probiotic strains to impact visceral fat depots [111].

Emerging RCTs of novel synbiotic products specifically targeting visceral adiposity further support these observations [112]. A recent 12-week double-blind, placebo-controlled trial showed that a synbiotic formulation significantly reduced visceral fat area, as quantified by Dual-Energy X-ray Absorptiometry (DXA), and modulated adipose distribution, with the strongest effects in men and in individuals with BMI in the overweight to class I obesity range [112]. Taken together, these trials suggest that although absolute weight loss attributable to probiotics and synbiotics is modest, central adiposity and visceral fat appear to be preferentially targeted, which is particularly relevant given their strong association with cardiometabolic risk [113]. Table 2 compares RCTs of probiotics and synbiotics for central adiposity and visceral fat, with differences in populations, formulations, duration, and outcomes.

### 4.4. Probiotics in Weight and Fat Metabolism Management

Clinical and experimental evidence indicates that probiotics primarily function as adjuncts in weight management rather than as standalone anti-obesity drugs, with the most robust benefits achieved by fine-tuning body composition and metabolic risk markers [114]. A recent review of probiotic use in excess weight concluded that in approximately two-thirds of RCTs, probiotic supplementation without formal diet therapy resulted in significant reductions in body weight and BMI, while about 80% of studies reported decreases in WC, and a subset showed improvements in total body fat mass [115]. These patterns are consistent with broader meta-analytic data demonstrating modest but consistent declines in weight, BMI, fat percentage, and WC with probiotic or synbiotic supplementation, particularly when multistrain preparations are used, and interventions extend beyond 8–12 weeks [96,116]. Recent glycemic benefits likely reflect combined effects on SCFA–GPR41/43 signaling, bile acid–FXR/TGR5 pathways, and barrier reinforcement that reduces LPS-driven inflammation. These overlapping mechanisms may explain why multistrain probiotics often improve HbA1c and HOMA-IR more than single strains.

Mechanistically, probiotics affect weight and fat metabolism via several interrelated pathways [114]. First, probiotics can indirectly support weight loss and weight maintenance by modulating gut-derived appetite hormones, such as peptide YY (PYY) and GLP-1, which reduce food intake, increase satiety, and modestly improve glycemic control by promoting SCFA production and FFAR2/3 signaling [117]. Second, probiotics modulate bile acid metabolism through bile salt hydrolase activity and changes in bile acid deconjugation, thereby affecting FXR and TGR5 signaling pathways that govern energy expenditure, hepatic gluconeogenesis, and lipid metabolism; this mechanism is consistent with observed improvements in hepatic steatosis and circulating lipid profiles across multiple trials [106]. Third, probiotics have barrier-protective and anti-inflammatory effects that reduce metabolic endotoxemia, including translocation of lipopolysaccharide (LPS) and other microbial components into the circulation, a key driver of adipose tissue inflammation and insulin resistance in obesity [118]. Probiotics reduce chronic low-grade inflammation and normalize adipokine secretion and insulin sensitivity by reinforcing tight junctions, increasing mucin secretion, and shifting microbial communities away from LPS-rich pathobionts, thus creating a metabolic milieu less favorable to fat accumulation and weight gain [118]. In patients with obesity and prediabetes or metabolic syndrome, changes in inflammatory markers and glycemic parameters frequently reflect the modest changes in body weight and fat distribution, emphasizing the role of immunometabolic modulation in probiotic-mediated benefits [114].

Recently published studies have strengthened the clinical relevance of probiotics in metabolic disease. A network meta-analysis of RCTs in adults with T2DM showed that multistrain probiotic combinations, particularly formulations containing *Lactobacillus*, *Bifidobacterium*, *Propionibacterium*, and *Streptococcus*, were among the most effective options for reducing fasting plasma glucose and HOMA-IR. At the same time, *Bifidobacterium*-based interventions ranked highest for HbA1c reduction. In obesity, newer dose–response and strain-specific studies suggest that *L. plantarum* and *L. fermentum* may exert benefits that depend on dose and duration, highlighting the importance of strain selection and intervention design. In NAFLD, recent randomized data on Huosheng Formula and related interventions further support the relevance of microbiota-targeted strategies for improving oxidative stress and hepatic lipid metabolism [119,120,121].

### 4.5. Mechanisms Influencing Body Weight and Fat Distribution

Reductions in WC, body fat, and insulin resistance likely reflect probiotic effects on satiety, inflammation, bile acid signaling, and barrier integrity. These outcomes appear to be downstream of the microbiota-related pathways discussed above. Beyond SCFA-driven HDAC inhibition and FIAF regulation, additional mechanisms have been implicated in probiotic effects on body weight and fat distribution [122]. Probiotic-induced shifts in gut microbial composition can alter the abundance of genes encoding carbohydrate-active enzymes and nutrient transporters, thereby changing the efficiency with which complex carbohydrates and lipids are digested and absorbed and modulating the net caloric extraction from the diet [123]. Interventions using human-origin probiotic cocktails report increased SCFA production and favorable shifts in the Firmicutes/Bacteroidetes ratio, which correlate with reductions in visceral adiposity and improvements in lipid and glucose metabolism, highlighting a connection between community-level functional reprogramming and regional fat distribution [106].

Probiotics also influence host endocrine and neuroendocrine response by altering SCFAs, bile acids, and aromatic microbial metabolites [122]. SCFAs and secondary bile acids signal enteroendocrine cells to regulate the release of GLP-1, PYY, and gastric inhibitory polypeptide, thereby affecting appetite, gastric emptying, and insulin secretion [122]. At the same time, indole derivatives and other tryptophan metabolites influence the circuits of the gut–brain axis involved in energy intake and expenditure [124]. These endocrine pathways help explain why probiotic interventions often blunt weight gain or stabilize body weight rather than causing large absolute losses, yet still confer clinically relevant benefits on central adiposity and cardiometabolic risk markers [114].

At the adipose-tissue level, probiotic-induced SCFAs and downstream signaling can promote browning of white adipose tissue, upregulate thermogenic genes, and increase mitochondrial biogenesis, thereby enhancing energy expenditure and shifting away from visceral fat accumulation [122]. Experimental models show that butyrate and propionate upregulate uncoupling proteins and adiponectin receptor expression in adipose tissue, changes associated with improved insulin sensitivity and reduced hepatic steatosis in HFD-fed rodents [122,125]. In parallel, microbiome-driven reductions in systemic inflammation, via lower LPS exposure and expansion of regulatory immune subsets such as Tregs and regulatory B cells, normalize adipose tissue immune tone, limiting pro-inflammatory macrophage infiltration and cytokine production that drive adipocyte hypertrophy and ectopic fat deposition [122].

Accumulating evidence also links probiotic supplementation with increases in taxa inversely associated with obesity and metabolic syndrome—notably *A. muciniphila* and selected *Bifidobacterium* and *Lactobacillus* species [125]. These microbes contribute to enhanced mucus layer integrity, beneficial bile acid profiles, and SCFA production, collectively influencing regional fat storage and systemic metabolic risk [125]. Clinical RCTs employing multistrain formulations containing *Bifidobacterium* and *Lactobacillus* species frequently report preferential reductions in visceral adipose tissue and liver fat content relative to subcutaneous adiposity, suggesting that microbiome modulation can selectively deplete metabolically harmful fat compartments even when total weight loss remains modest [114].

Taken together, current evidence indicates that probiotics and synbiotics exert a multifaceted influence on obesity, acting through SCFA-mediated epigenetic modulation of adipose tissue, regulation of FIAF and energy harvest, modulation of endocrine and bile acid signaling, reinforcement of barrier integrity, and recalibration of immunometabolic pathways [122]. While average reductions in BMI and body weight are modest, generally in the range of 0.5–1.0 kg m^−2^ and approximately 1 kg, respectively, sustained improvements in central adiposity, visceral fat burden, glycemic control, and inflammatory status position probiotics as promising adjunctive agents within comprehensive obesity-management strategies that integrate dietary modification, physical activity, and pharmacologic therapies [122]. On the other hand, earlier meta-analyses, particularly those pooling small, short-duration RCTs, have reported non-significant or clinically trivial changes in body weight and BMI, with substantial between-study heterogeneity and notable risks of selective reporting and attrition bias, limiting the certainty of any conclusions about probiotic-induced weight loss [96,126,127].

## 5. Impact on Diabetes

### 5.1. Clinical Trials and Glycemic Outcomes

RCTs and meta-analyses in adults with T2DM consistently show modest improvements in glycemic control with probiotic supplementation, especially with multistrain, high-dose formulations administered for 8–12 weeks. Meta-analyses of 15 RCTs (*n* ≈ 900) indicated significant reductions in HbA1c, fasting blood glucose (FBG), and HOMA-IR versus baseline in T2DM patients treated with probiotic preparations. Still, effect sizes were small and heterogeneous [128]. A more recent network-style synthesis and dose–response analysis also found that probiotics reduced HbA1c by about 0.3–0.4 percentage points and improved HOMA-IR, with longer intervention duration (≥12 weeks) and higher daily doses associated with greater benefit [129]. These benefits likely reflect strain-dependent differences in SCFA signaling, bile acid remodeling, and barrier reinforcement rather than a uniform probiotic effect. The clinical effects of probiotics are strain-specific rather than interchangeable. Multistrain formulations may provide broader benefits than single strains because they combine complementary mechanisms, including SCFA generation, bile acid remodeling, epithelial barrier reinforcement, and immunometabolic modulation. Accordingly, the observed reductions in HbA1c and HOMA-IR should be interpreted as the downstream result of these strain-dependent biological actions rather than as a class effect of all probiotics [130,131].

Several RCTs in T2DM have tested probiotic yogurts or fermented milks containing *Lactobacillus* and *Bifidobacterium* species, demonstrating reductions in FBG, postprandial glucose, and fasting insulin, and improved antioxidant status [30]. For example, probiotic yogurt containing *L. acidophilus* La5 and *B. animalis* subsp. lactis Bb12 (300 g/day for 6 weeks) decreased FBG and increased total antioxidant capacity in T2DM subjects compared to conventional yogurt [30]. Other studies with multistrain probiotic capsules (e.g., *L. acidophilus*, *L. casei*, *L. lactis*, *B. bifidum*, *B. longum*, and *B. infantis* in combination) have shown improvements in HbA1c and fasting insulin without any change in body weight, suggesting direct glycemic effects not entirely mediated by weight loss [132].

**Figure 4:**
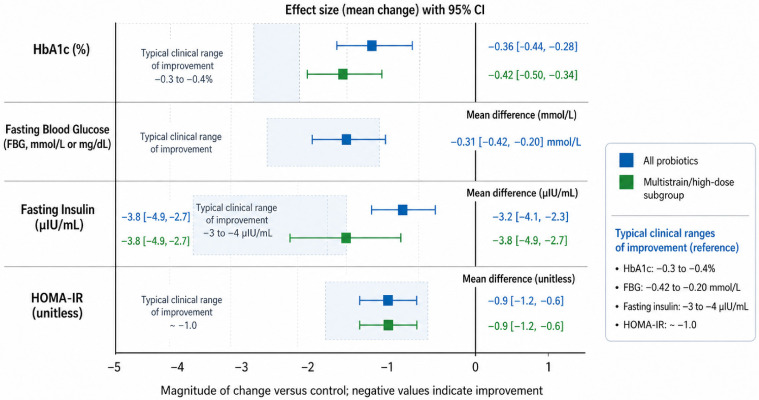
Core glycemic outcomes after probiotic and synbiotic interventions. Blue bars represent pooled estimates from all probiotic interventions across meta-analyses, and green bars represent multistrain and/or higher-dose subgroup estimates. Negative values indicate reductions relative to the control, and whiskers show 95% confidence intervals. Typical effect sizes are shown for HbA1c, fasting glucose, fasting insulin, and HOMA-IR. These glycemic improvements are consistent with the barrier-reinforcing and anti-inflammatory actions of probiotics described in earlier sections, in which reduced intestinal permeability and LPS translocation contribute to lower systemic inflammation and improved insulin sensitivity. Figure was created using the FigureLabs AI platform (https://www.figurelabs.ai/).

However, not all syntheses are equally positive: a meta-analysis in 2022 found that while probiotics did not lead to clinically significant HbA1c reductions, they did result in modest but statistically significant reductions in FBG and fasting insulin, especially with multistrain, high-dose regimens and in patients with higher baseline BMI [128]. More recent analyses of probiotic combinations have confirmed significant reductions in HOMA-IR and fasting glucose but have also highlighted the heterogeneity of strains, doses, and background therapies, warning that effect sizes are often smaller than those seen with first-line pharmacotherapy and that probiotics should be considered as adjuncts, not standalone treatments [133]. Figure 4, as detailed in the mechanisms section, many of these modest but consistent improvements in HbA1c, fasting glucose, fasting insulin, and HOMA-IR are downstream of restored intestinal barrier integrity and reduced metabolic endotoxemia: probiotic-induced upregulation of tight junctions and mucus, together with reductions in LPS-driven NF-κB activation, attenuate systemic inflammation, thereby improving insulin sensitivity and β-cell function in obesity and T2DM.

### 5.2. Mechanistic Basis of Probiotic Effects on Diabetes

Probiotic action in diabetes is mechanistically multifactorial: it encompasses modulation of microbial ecology, reinforcement of intestinal barrier integrity, attenuation of metabolic inflammation, and remodeling of SCFAs, bile acid, and indole signaling pathways that converge on insulin sensitivity and β-cell function [98]. Dysbiosis in T2DM is characterized by reduced diversity, depletion of SCFA-producing taxa (e.g., Faecalibacterium, Roseburia, Akkermansia), and enrichment of pathobionts that increase luminal lipopolysaccharide (LPS) and branched-chain amino acids, thereby promoting metabolic endotoxemia and insulin resistance via toll-like receptor (TLR)–NF-κB signaling [134]. Probiotic and synbiotic interventions partially restore the abundance of beneficial commensals, increase SCFA production, and reduce LPS translocation, collectively diminishing systemic low-grade inflammation [13,14].

SCFAs, such as acetate, propionate, and butyrate, act on G-protein-coupled receptors (GPR41), G-Protein Coupled Receptor 43 (GPR43), and G-Protein Coupled Receptor 109A (GPR109A) on enteroendocrine, immune, and adipose cells to enhance GLP-1 secretion, improve insulin signaling, and promote regulatory immune phenotypes [125]. Experimental SCFA “biotherapy” in humans and humanized mice indicates that increasing colonic SCFA remodels the mucosal proteome and metabolome, enriches anti-inflammatory pathways, and delays diabetes progression, with fecal transplantation from SCFA-responders transferring protection to gnotobiotic mice [135]. Similarly, SCFA cocktails in HFD-induced diabetic mice improve insulin signaling, upregulate GLUT4 and PPARα, modulate hepatic lipogenesis (SREBP1c and LXR), and increase expression of SCFA receptors, while restoring butyrate-producing taxa such as Roseburia and Lachnospiraceae [14].

Bile acid signaling constitutes another mechanistic node: microbial deconjugation and transformation influence the activation of the FXR and Takeda G-protein receptor 5 (TGR5), thereby modulating GLP-1 secretion, hepatic gluconeogenesis, and energy expenditure [136]. Probiotics and next-generation strains, such as *A. muciniphila*, alter primary/secondary bile acid ratios and promote FXR–FGF19 signaling, thereby improving insulin sensitivity and hepatic lipid handling [72]. In parallel, probiotics dampen NF-κB-driven inflammatory cascades in intestinal and adipose tissues, reduce circulating TNF-α and IL-6, increase IL-10 and TGF-β, and enhance regulatory T-cell (Treg) function, collectively ameliorating insulin resistance and β-cell stress [137].

### 5.3. L. casei Plus Inulin

Synbiotic combinations of *L. casei* with inulin have been explored as a strategy to combine direct probiotic effects with fermentation of a selective prebiotic substrate, thereby increasing SCFA production and supporting engraftment [14,138]. In a randomized trial in T2DM patients, *L. casei* supplementation (10^8^ CFU/day for 8 weeks) improved glycemic response and modulated serum sirtuin-1 (SIRT1) and fetuin-A levels; SIRT1 upregulation and fetuin-A reduction were proposed as mechanistic links between probiotic use and improved insulin sensitivity [139]. Preclinical work in HFD/streptozotocin models indicates that co-administration of *Lactobacillus* strains with inulin alleviates cardiac oxidative stress, reduces caspase activation, and improves insulin and fasting glucose levels by modulating leptin receptor expression and antioxidant enzyme activity [140,141].

Synbiotic supplementation significantly improved insulin metabolism markers (reduced insulin, HOMA-IR, HOMA-β; increased QUICKI) and lipid profiles (lowered TAG and VLDL-cholesterol) in women with gestational diabetes after 6 weeks, compared to placebo [142]. In pregnant women with gestational diabetes, a synbiotic containing *L. sporogenes* plus inulin improved HOMA-IR and fasting insulin, although effects on FBG were limited [142]. Another trial using a synbiotic capsule comprising *L. acidophilus*, *L. casei*, *B. bifidum*, and inulin in gestational diabetes demonstrated reductions in insulin and HOMA-IR over 6–8 weeks, supporting the use of *L. casei*-based synbiotics in hyperglycemic states [142].

**Figure 5:**
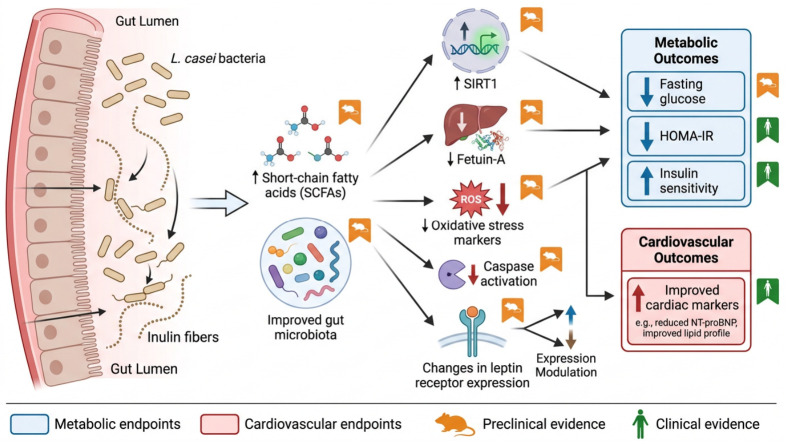
*L. casei*–inulin synbiotics improve microbiota, SCFA production, and SIRT1 signaling while reducing fetuin-A, oxidative stress, and caspase activation, leading to better metabolic and cardiovascular outcomes. Orange icons denote preclinical evidence, green icons denote clinical evidence, blue boxes mark metabolic endpoints, and red boxes mark cardiovascular endpoints. Figure was created using the FigureLabs AI platform (https://www.figurelabs.ai/).

Although most synbiotic data are from non-diabetic or mixed populations, mechanistic coherence across models suggests that *L. casei*–inulin combinations reduce oxidative stress, modulate adipokines (fetuin-A, leptin), and improve insulin signaling, with potential translatability to T2DM and prediabetes [14]. Future work should prioritize well-powered RCTs in T2DM with metagenomic and metabolomic endpoints to define responders and optimize dose and matrix [72]. Interestingly, Figure 5 shows how *L. casei*–inulin synbiotics may improve metabolic and cardiovascular health by reshaping the gut microbiota, increasing SCFAs, and modulating SIRT1, fetuin-A, oxidative stress, caspase activation, and leptin receptor expression. These changes are associated with improved glycemic control and reduced cardiac markers in T2DM and gestational diabetes.

### 5.4. Glycemic Control and Insulin Sensitivity

Beyond single trials, pooled analyses show that probiotics modestly improve insulin sensitivity, as reflected in HOMA-IR, fasting insulin, and postprandial glucose excursions [133]. In meta-analyses of RCTs in T2DM, probiotic supplementation reduced HOMA-IR by approximately 1 unit and lowered fasting insulin versus controls, with multistrain formulations achieving greater effects than single strains [133]. A 2024 meta-analysis focusing on probiotic combinations reported significant reductions in HOMA-IR (MD ≈ −1.05) and fasting insulin (≈−3–4 µIU mL^−1^), along with small decreases in fasting glucose [143]. Network meta-analysis ranking interventions suggests that multistrain, high-dose probiotics and synbiotics occupy the upper tiers for improving HbA1c and insulin resistance, although heterogeneity and risk of bias limit firm comparative conclusions [144].

Clinical studies using probiotic yogurts and fermented milks demonstrate improvements in FBG and HbA1c without changes in BMI, indicating that microbiome-mediated mechanisms can operate independently of weight loss [30,145,146]. For example, probiotic fermented milk containing *L. acidophilus* and *B. lactis* reduced FBG and HbA1c over 8 weeks in T2DM patients compared with conventional fermented milk, accompanied by modest improvements in lipid profile and antioxidant status [147,148]. Similarly, a five-strain probiotic formulation (WBF-011) enhanced postprandial glucose control (reduced glucose Area Under the Curve (AUC)) in adults with T2DM on metformin without significant changes in fasting glucose or weight, suggesting specific effects on postprandial insulin sensitivity and incretin response [132]. Collectively, these findings indicate that probiotics can achieve clinically modest improvements in glycemic indices and insulin sensitivity, particularly when multistrain, high-dose formulations are used for at least 12 weeks and administered alongside standard lifestyle and pharmacologic therapy [129,149]. Importantly, effects are heterogeneous and appear to be larger in those with higher baseline dysglycaemia and BMI, highlighting the importance of a stratified, precision-oriented approach in future trials [149].

On the other side, meta-analyses in adults with type 2 diabetes and related dysglycemia indicate that probiotic or synbiotic supplementation produces modest reductions in fasting glucose and HOMA-IR, with small decreases in fasting insulin and HbA1c. Effect sizes are heterogeneous and often hover near the lower bound of clinical relevance. Some pooled estimates for fasting glucose or HbA1c are not significant, so probiotics are best regarded as adjuncts that may offer modest benefits in selected populations rather than stand-alone glycemic therapies [150,151,152].

### 5.5. Additional Mechanisms and Metabolic Pathways

In addition to SCFA and bile acid signaling, probiotics influence multiple metabolic pathways relevant to diabetes, including tryptophan–indole pathways, branched-chain amino acid (BCAA) metabolism, and redox balance [125]. Indole derivatives produced by commensal bacteria modulate aryl hydrocarbon receptor (AhR) signaling to reinforce barrier integrity and modulate mucosal immunity; SCFA-enriched microbiota after biotherapy increased AhR ligands and IgA production, which were associated with delayed diabetes progression in NOD mice colonized with responder microbiota [153]. Probiotics can also reduce circulating BCAAs and aromatic amino acids, which have been linked to insulin resistance, by shifting microbiome composition toward taxa that preferentially ferment fibers into SCFAs rather than proteolytic metabolites [154].

Emerging evidence implicates direct modulation of enteroendocrine and β-cell function by next-generation probiotics and their postbiotic fractions [72]. In vitro work shows that *A. muciniphila* extracts stimulate GLP-1 secretion in human L-cells and increase glucose-stimulated insulin secretion in β-cell lines [72]. These effects may translate into modest improvements in glycemic control in vivo [72]. A first-in-human RCT of pasteurized *A. muciniphila* in overweight/obese adults with insulin resistance demonstrated improved insulin sensitivity (+28%), reduced fasting insulin (−34%), and decreased total cholesterol compared with placebo, supporting a role for this species as a metabolic modulator [72]. Engineered probiotics have also been developed to release GLP-1–modulating peptides in the intestine, normalizing barrier integrity and improving metabolism in fiber-deprived mice, illustrating the potential of synthetic biology to harness probiotic–endocrine crosstalk for diabetes therapy [30]. Oxidative stress and metaflammation represent additional targets: probiotic and synbiotic interventions have been shown to upregulate antioxidant enzymes (SOD, CAT, GPx), reduce lipid peroxidation, and decrease systemic inflammatory markers in diabetic models and patients [30]. These redox and inflammatory effects likely intersect with insulin signaling and β-cell preservation, providing a broader mechanistic context for the operation of SCFAs, bile acids, and indole pathways [98].

Notably, in several trials, improvements in BMI, WC, and glycemic indices were accompanied by reductions in circulating MDA and oxidized LDL, and by increases in total antioxidant capacity and in SOD and GPx activities, supporting a mechanistic link between probiotic supplementation, redox balance, and metabolic outcomes.

## 6. Innovative Strategies in Management

### 6.1. Synbiotics, Engraftment Support, and Postbiotics

Synbiotics, combinations of probiotics with complementary prebiotics, are increasingly used to enhance engraftment, metabolic activity, and persistence of administered strains in the hostile gastrointestinal environment [125]. Systematic reviews in NAFLD show that synbiotics can improve liver enzymes, steatosis scores, and some metabolic parameters, although effects on glycaemia and insulin resistance are inconsistent across small heterogeneous RCTs [86,155]. Individual trials indicate that synbiotic capsules containing *Lactobacillus*/*Bifidobacterium* strains plus inulin or fructooligosaccharides improve HOMA-IR, inflammatory markers, and lipid profiles in gestational diabetes and metabolic syndrome, suggesting that prebiotic co-administration strengthens probiotic functional impact in metabolic disease [142].

Postbiotics, non-viable microbes or their components/metabolites, represent a clinically attractive alternative for metabolic indications in which safety, stability, and regulatory simplicity are priorities [156]. SCFA-yielding biotherapies and defined SCFA mixtures have been administered orally to adults with type 1 diabetes and to HFD-induced diabetic mice, increasing fecal and plasma SCFA levels, reshaping the gut microbiota, improving liver metabolism, and delaying diabetes progression, without the survival challenges of live probiotics [157]. Pasteurized *A. muciniphila*, which is non-replicating but retains an intact outer membrane, improved insulin sensitivity and cardiometabolic parameters in overweight/obese adults, demonstrating that heat-killed or pasteurized preparations can function as next-generation postbiotics with enhanced stability and safety [72].

### 6.2. Microbiome-Engineered Consortia and AI-Enabled Personalization

Microbiome-engineered consortia, defined communities of strains with complementary metabolic capabilities, represent a next step beyond single-strain or empirical multistrain formulations [158,159]. Proof-of-concept work adapting *Faecalibacterium prausnitzii* to tolerate oxygen exposure has enabled the development of a symbiotic product compatible with manufacturing and human consumption, with early clinical testing demonstrating colonization in a subset of participants and laying foundations for oxygen-tolerant next-generation probiotics targeting inflammatory and metabolic disorders [160,161]. Similar approaches can assemble consortia that jointly produce SCFAs, secondary bile acids, and indoles, or that occupy metabolic niches depleted in T2DM and NAFLD [162].

Artificial intelligence (AI) and machine learning now integrate multi-omic microbiome, dietary, and clinical data to predict glycemic response and identify probiotic candidates and microbial signatures associated with prediabetes and obesity [163]. AI-empowered microbiome research has begun to generate personalized nutrition algorithms and strain-selection pipelines that outperform traditional methods in predicting postprandial glycemic response and in screening large probiotic libraries for desirable metabolite profiles [163]. These tools are expected to underpin precision probiotic prescriptions, matching specific consortia or NGPs to individual dysbiosis and metabolomic signatures in T2DM and obesity [162].

### 6.3. Fermented Foods, Fecal Microbiota Transplantation (FMT), and Adjunctive Modalities

Fermented dairy products, particularly yogurts containing live cultures, occupy a pragmatic interface between traditional foods and probiotic interventions [164,165]. Large prospective cohorts and meta-analyses indicate that higher intake of fermented dairy products is associated with a lower risk of incident T2DM, with yogurt consumption ≥ 2 servings per week linked to roughly 40–60% lower risk than minimal intake [166]. Mechanistically, fermented dairy provides probiotics, prebiotic substrates, and bioactive peptides and vitamins (e.g., vitamin K2) that collectively modulate microbiota composition, inflammation, and insulin sensitivity, and these foods are foundational components of Mediterranean-style patterns associated with reduced cardiometabolic risk [163].

FMT offers a more radical approach to modulating the microbiome in diabetes [167,168]. Experimental work in db/db mice shows that FMT from healthy donors improves FBG, insulin sensitivity, intestinal barrier function, and inflammatory profiles, accompanied by concomitant shifts in the microbiota and serum metabolites [162]. Prior human pilot studies also suggest that FMT from lean donors can transiently improve insulin sensitivity in individuals with metabolic syndrome. However, durability and safety remain concerns, and FMT is currently reserved for refractory *Clostridioides difficile* infection rather than metabolic disease [169]. Conceptually, insights from FMT studies are informing the design of rational consortia and the selection of responder-like donor microbiomes as templates for engineered probiotics [162].

### 6.4. GLP-1 Agonist Combinations and Endocrine Targeting

The widespread use of GLP-1 receptor agonists and dual/triple incretin mimetics in obesity and T2DM raises the question of how to integrate microbiome-targeted interventions [170,171]. Engineered probiotics that locally elevate GLP-1 in the intestine have been shown to restore barrier integrity and improve metabolic parameters in fiber-deprived mice, offering an endogenous means of incretin modulation that may complement pharmacologic GLP-1 receptor agonists [172]. Preclinical work suggests that such engineered strains could reduce required drug doses, mitigate gastrointestinal side effects, or maintain GLP-1 signaling during treatment breaks, though human data are still lacking [172].

In parallel, Akkermansia-based preparations and SCFA biotherapies increase GLP-1 secretion and downstream insulinotropic effects, suggesting multimodal strategies in which GLP-1 receptor agonists, NGPs, and SCFA/postbiotic formulations act in concert on the enteroendocrine-insulin axis [72,82]. Future clinical trials will need to investigate pharmacokinetic and pharmacodynamic interactions, potential effects on appetite and weight loss trajectories, and the impact of GLP-1-induced changes in gastric emptying on probiotic engraftment [162].

### 6.5. Novel Intervention Approaches and Future Directions

Current research trends are to incorporate probiotics into dietary patterns and prebiotic matrices rather than deliver isolated strains, in line with ecological principles of microbiome resilience [163]. Mediterranean-style diets rich in whole grains, legumes, fruits, vegetables, nuts, olive oil, and fermented dairy products contain fermentable fibers and polyphenols that favor SCFA-producing taxa and can synergize with probiotic supplementation to ameliorate insulin sensitivity and reduce the risk of T2DM [163]. The combination of multistrain probiotics with tailored fibers (inulin, resistant starch, arabinoxylans) and polyphenol-rich foods (berries, pomegranate, tea) is being investigated to potentiate SCFA production, bile acid remodeling, and antioxidant effects in metabolic disease [163].

Emerging avenues include time-restricted or circadian-aligned probiotic dosing to align with host metabolic rhythms [173,174]. These microbiome-responsive delivery systems release probiotics or postbiotics in response to pH or metabolite cues, and adjunctive use of exercise and sleep interventions known to reshape the microbiome in ways favorable for insulin sensitivity [174]. Ultimately, precision strategies will likely employ baseline metagenomic, metabolomic, and clinical data to stratify individuals into responder subtypes and allocate specific consortia, postbiotics, or diet–probiotic packages beyond one-size-fits-all supplementation [175,176].

## 7. Clinical Applications and Recommendations

### 7.1. Dosing, Formulations, and Treatment Duration

Effective doses of probiotics in RCTs for T2DM and metabolic syndrome are generally 10^9^–10^11^ CFU/day and are usually administered as capsules or fermented dairy products [149]. Higher doses and multistrain formulations (≥2–3 strains) seem to offer more robust glycemic benefits than single-strain, low-dose preparations, although an exact dose–response curve remains elusive [20,119]. A pragmatic starting recommendation from a clinical standpoint is 10^9^–10^10^ CFU/day of a multistrain preparation containing *Lactobacillus* and *Bifidobacterium* species with documented survival and metabolic activity in the human gut [119,177].

The majority of improvements in glycaemia and insulin sensitivity seem to occur after 8–12 weeks, and some meta-analyses have suggested that interventions lasting 12–24 weeks produce larger reductions in HbA1c and HOMA-IR [119,129]. Thus, an initial treatment duration of 12–24 weeks appears reasonable to assess response, followed by maintenance dosing, which may be continuous or intermittent based on patient preference, adherence, and stability of metabolic control [129]. Fermented dairy vehicles (e.g., yogurt, kefir) offer a patient-friendly delivery mode that also contributes nutrients and bioactive peptides, and have supportive epidemiologic evidence for lowering T2DM risk when consumed regularly [178,179].

### 7.2. Tailoring to Dysbiosis Profiles and NAFLD/T2D Phenotypes

Given substantial inter-individual variability in microbiome composition and probiotic response, tailoring interventions to dysbiosis profiles is an emerging priority [84,180]. NAFLD and T2DM are typically associated with reduced abundance of SCFA-producing taxa (Faecalibacterium, Roseburia, Akkermansia) and enrichment of LPS-producing Gram-negative bacteria; FMT and SCFA-biotherapy studies confirm that restoring SCFA production and barrier integrity can improve insulin sensitivity and hepatic steatosis [86,181,182]. Synbiotic RCTs in NAFLD show improvements in liver enzymes and indices of steatosis [183]. However, glycemic effects are variable, suggesting that targeting hepatic and intestinal inflammation may be particularly relevant in NAFLD-predominant phenotypes [86].

In practice, clinical integration may proceed by using stool-based microbiome profiling or simpler proxies (e.g., high triglycerides, elevated liver enzymes, central adiposity, and low fiber intake) to infer likely deficits in SCFA-producing bacteria and guide the selection of probiotic–prebiotic combinations that emphasize SCFA-generating strains and substrates [83]. For example, in patients with NAFLD/T2D and evidence of barrier dysfunction, combinations including *Lactobacillus*, *Bifidobacterium*, and *Akkermansia* (or *Akkermansia*-derived postbiotics) alongside inulin and resistant starch may be prioritized to enhance barrier integrity, reduce endotoxemia, and improve hepatic insulin sensitivity [85,184].

### 7.3. Lifestyle Integration and Synergy with Mediterranean Patterns

Probiotics should be embedded within broader lifestyle interventions rather than used in isolation [185,186]. Mediterranean-style dietary patterns, which include abundant plant fibers, polyphenols, and fermented dairy products, are consistently associated with lower incidence of T2DM and improved cardiovascular outcomes, with mechanistic links to increased microbial diversity, higher SCFA levels, and reduced inflammatory tone [186,187,188]. Consumption of fermented dairy within a Mediterranean pattern appears to partially mediate the benefit, with yogurt intake specifically associated with lower T2DM risk in cohort studies [186]. Non-nutritive sweeteners may help lower energy intake and postprandial glycemia. Still, emerging evidence suggests that some products can deplete beneficial taxa, promote pro-inflammatory species, and disrupt SCFA-linked metabolic pathways, warranting cautious use and careful evaluation of their long-term effects on the microbiome-mediated metabolism [189].

Clinically, integrating probiotic-rich foods (yogurt, kefir, traditional fermented vegetables) and/or supplements into Mediterranean-aligned diets, combined with structured physical activity and sleep hygiene, may maximize microbiome-mediated benefits for glycemic control and cardiometabolic risk reduction [186,190]. Patient education should emphasize that probiotics are adjunctive tools that work best when layered onto dietary, exercise, and pharmacologic strategies, and that consistency over months is essential to maintain microbiome shifts and metabolic gains [185,191].

Notably, Table 3 condenses practical probiotic and synbiotic recommendations for obesity, T2DM, and NAFLD/T2DM, linking formulation, dose, duration, and co-interventions to the relevant clinical phenotype. It also bridges the mechanistic and trial evidence to a more usable clinical framework.

## 8. Challenges and Future Perspectives

### 8.1. Strain Specificity, Response Variability, and Safety

A central challenge in translating probiotic science to clinical diabetes care is pronounced strain specificity: benefits observed with a given strain or formulation cannot be extrapolated to other products, even within the same species [131,192]. Meta-analyses reveal high heterogeneity across trials, reflecting differences in strains, doses, matrices, baseline microbiota, background medications, and adherence, with some large syntheses concluding that while average effects on fasting glucose and insulin are modestly beneficial, HbA1c reductions may not reach conventional thresholds of clinical significance in unselected T2DM populations [150,193]. Interindividual variability is also driven by baseline dysbiosis patterns and host genetics, highlighting the need for responder-focused designs and stratified analyses [194,195,196].

Efficacy is also limited by the need to survive gastric acid and bile, as well as by engraftment and persistence, especially for oxygen-sensitive next-generation strains such as *F. prausnitzii* and *A. muciniphila*. Technologies for encapsulation, oxygen adaptation, and synbiotic co-delivery are beginning to address these challenges but add complexity to manufacturing and regulation [38,197,198,199,200]. In addition, numerous microbiome changes observed after supplementation are transient, disappearing after stopping the probiotic, implying that lasting benefits may require continuous intake and favorable dietary patterns [201,202].

Safety is generally favorable in immunocompetent adults, with large reviews and clinical experience indicating low rates of serious adverse events [201,202]. However, rare cases of bacteremia and fungemia due to probiotic organisms (*Lactobacillus*, *Bacillus clausii*, *Saccharomyces boulardii*) have been documented, predominantly in critically ill, immunocompromised, or catheterized patients, and matched case–control data indicate markedly increased odds of probiotic exposure among patients with invasive infections by typical probiotic species [201,203,204]. Consequently, guidelines advise caution or avoidance of live probiotics in severely immunocompromised individuals, those with central venous catheters, or those with uncontrolled critical illness, while engineered, safer strains and postbiotics are under development for these populations [205,206,207].

### 8.2. Methodological Gaps and Trial Design Needs

Most probiotic RCTs in T2DM and metabolic syndrome are small, short, and underpowered for hard endpoints, often lacking detailed microbiome, metabolome, or immune phenotyping, which limits mechanistic interpretation and identification of responders [33,150,208]. Heterogeneity in strain identity, dose, matrix, and outcome measures hampers meta-analytic synthesis and the ability to generate strain- or product-specific clinical recommendations [26,193,208]. Longitudinal, multi-center RCTs with 6–24-month follow-up, standardized metabolic endpoints, and harmonized microbiome and metabolomic protocols are needed to clarify the durability of effects, impact on complications, and interactions with pharmacotherapies such as metformin, SGLT2 inhibitors, and GLP-1 receptor agonists [33,209].

Future trials should incorporate baseline and on-treatment metagenomic sequencing, targeted and untargeted metabolomics (SCFAs, bile acids, indoles, BCAAs), and immune profiling to derive mechanistically anchored responder signatures and to test whether specific microbiome changes mediate clinical benefits [210,211]. Adaptive designs and N-of-1 trials may be useful in tailoring interventions to individual microbiome configurations, particularly when testing microbiome-engineered consortia and NGPs [212,213].

### 8.3. Next-Generation Probiotics, Biomarkers, and Regulation of Postbiotics

Next-generation probiotics such as *A. muciniphila* and *F. prausnitzii*, along with SCFA-yielding biotherapies, are advancing through early-phase clinical development, with initial data supporting safety and metabolic benefits but with many questions remaining regarding optimal dosing, long-term safety, and efficacy across diverse patient populations [79,94,214,215]. Biomarkers such as fecal SCFA levels, bile acid profiles, and specific microbial taxa or gene signatures are being investigated to identify responders and monitor biological activity, with SCFA elevations and the restoration of butyrate producers emerging as promising indicators of therapeutic engagement [35,215,216,217]. Worth noting, *A. muciniphila* emerges as a promising next-generation candidate whose clinical translation requires further confirmation in well-designed, long-term trials.

Regulatory frameworks have yet to fully keep pace with postbiotics and NGPs, which are classified as dietary supplements, novel foods, and drugs in different jurisdictions [218,219,220]. Authorities will need to define standards for characterization, manufacturing, and clinical evidence, including requirements for strain-level identification, genomic stability, absence of mobile antibiotic resistance genes, and demonstration of safety in vulnerable groups [219,221,222]. For postbiotics, clear guidance on compositional consistency and mechanism-of-action claims will be essential to prevent over-marketing of poorly characterized preparations [223,224]. Table 4 summarizes the key barriers to translating probiotic science into metabolic care and the practical approaches that may overcome them. It also bridges the evidence on emerging probiotic strategies with the broader translational challenges that still need to be addressed.

From a translational standpoint, the development of next-generation probiotics and postbiotics faces substantial regulatory and industrial hurdles. In Europe, strains such as pasteurized *A. muciniphila* are evaluated under the Novel Food framework, which requires comprehensive safety dossiers, specification of intended use and target populations (often limited to adults with overweight or obesity), and strict control of manufacturing processes [225]. In the United States, live or inactivated strains and purified postbiotic components may require GRAS notification or other regulatory pathways, depending on whether they are marketed as foods, dietary supplements, or drugs [226,227]. In addition, many candidate strains are obligate anaerobes with limited intrinsic stability, making large-scale production, formulation, and cold-chain distribution challenging. Strategies such as pasteurization, encapsulation, lyophilization, and incorporation into robust food matrices seek to balance strain viability or activity with shelf-life and safety. Still, they must be validated for each strain and formulation [228,229]. Finally, custom-made bacterial mixtures and precision consortia raise additional complexity in terms of quality control, strain identification, batch consistency, and regulatory classification, which currently limit their widespread use beyond clinical trials.

## 9. Conclusions and Remarks

Accumulating evidence from clinical trials and mechanistic studies supports a role for probiotics and related microbiome-targeted strategies as adjunctive tools in the management of obesity and diabetes [33,94]. In T2DM, multistrain probiotic and synbiotic interventions at doses of 10^9^–10^10^ CFU/day over 12–24 weeks modestly improve HbA1c, fasting glucose, and insulin resistance, particularly in individuals with pronounced dysglycaemia and higher BMI, while fermented dairy consumption is consistently associated with a lower incidence of T2DM in observational cohorts [33,129,230]. These clinical effects are underpinned by convergent mechanistic pathways, including restoration of SCFA production, remodeling of bile acid and indole signaling, reinforcement of barrier integrity, and dampening of NF-κB-driven metaflammation, which together enhance insulin sensitivity and support β-cell function [33,34,35].

Innovation is rapidly extending beyond conventional probiotics to synbiotics optimized for engraftment, postbiotic preparations such as pasteurized *A. muciniphila* and SCFA-biotherapies, engineered consortia enriched in NGPs, and AI-guided personalization frameworks that match specific microbial interventions to individual dysbiosis and metabolic signatures [214]. These developments are part of the broader evolution toward precision, multimodal treatment of metabolic disease, combining microbiome-targeted therapies with GLP-1 receptor agonists, lifestyle modification, and cardiometabolic pharmacotherapy for sustained risk reduction [192,195].

At the same time, there are major challenges in strain specificity, significant inter-individual variability, a limited number of long-term RCTs with mechanistic endpoints, and safety in immunocompromised populations [192,230]. Future research should prioritize large, longitudinal, metagenomics-enabled RCTs of both conventional and next-generation probiotics; rigorous evaluation of biomarkers, such as fecal SCFA and bile acid signatures, for identifying responders; and the development of coherent regulatory frameworks for postbiotics and NGPs [195,216]. Within such a framework, personalized, microbiome-informed probiotic strategies have the potential to complement and extend pharmacological approaches, helping to re-establish eubiosis and to address the immunometabolic roots of obesity and diabetes beyond what glucose-lowering drugs alone can achieve [33,35].

In conclusion, this review demonstrates that probiotics, through mechanisms such as SCFA production, bile acid remodeling, barrier reinforcement, and immune modulation, offer modest yet consistent benefits in managing obesity and T2DM, including reductions in BMI (0.3 kg m^−2^), WC (1–2 cm), HbA1c (0.3–0.4%), and HOMA-IR. These findings underscore the microbiome’s pivotal role in immunometabolic health, positioning strain-specific probiotics such as *L. rhamnosus* GG, *B. breve*, and *A. muciniphila* as valuable adjuncts to lifestyle and pharmacotherapy, particularly in dysbiosis-driven conditions such as NAFLD. Future longitudinal RCTs with metagenomic and metabolomic endpoints, alongside innovations in synbiotics, postbiotics, and AI-tailored consortia, will refine precision strategies to achieve durable cardiometabolic improvements.

## Figures and Tables

**Table 1:** Strain-specific mechanistic signatures of key probiotic and next-generation strains.

Strain (Species + Strain ID)	Model/Population	Primary Mechanistic Targets	Key Metabolites/Signals	Main Metabolic Outcomes	Ref.
Multistrain/synbiotic formulations	Obese adults; T2DM; metabolic syndrome; gestational diabetes	Community-level remodeling, SCFA support, barrier support, immune modulation, and engraftment enhancement	SCFAs, GLP-1, PYY, cytokines, bile acids, Treg/NF-κB	↓ BMI, ↓ WC, ↓ HOMA-IR, ↓ fasting glucose, improved insulin sensitivity	[14,48,78,79,80]
*A. muciniphila* (live or pasteurized)	HFD models; obesity/T2DM translational studies	Gut–liver axis, mucin repair, FXR–FGF15 axis, SCFA-related signaling	Propionate, butyrate, FXR/FGF15, GLP-1	↓ visceral adiposity, ↓ fatty liver, ↑ glucose control	[55,81]
*B. breve* plus *L. plantarum*	Obese adults	Central adiposity modulation, microbiota remodeling, visceral/subcutaneous fat redistribution	SCFAs, inflammatory markers, microbiota shifts	↓ WC, ↓ visceral-to-subcutaneous fat ratio	[57]
*L. rhamnosus* GG (LGG)	Human enteroids/colonoids; epithelial monolayers; in vivo barrier studies	Tight junction reinforcement, barrier protection, ZO-1 and occludin preservation, reduced NF-κB activation, and mucus-layer support	↑ ZO-1 and occludin; ↑ mucin; ↓ NF-κB p65 nuclear translocation; ↓ CXCL8; ↓ endotoxin/LPS translocation	↓ intestinal permeability, ↓ endotoxin translocation, ↓ metabolic inflammation, improved barrier integrity	[63,64]
*B. breve* BBr60	Obese adults; prediabetic adults; microbiota/metabolome studies	Cross-feeding-mediated SCFA production, especially butyrate; modulation of GLP-1 and IL-27; reduction in IL-1β	↑ fecal butyrate; ↑ GLP-1 and IL-27; ↓ IL-1β; SCFA-linked modulation of inflammatory and hormonal signaling	↓ body weight, ↓ body fat %, ↓ HOMA-IR, ↑ glycemic control, ↓ inflammation	[66,67]
Pasteurized *A. muciniphila*	Overweight/obese adults with insulin resistance	Barrier and metabolic signaling, bile acid and enteroendocrine modulation, improved insulin sensitivity	GLP-1, bile acids, hypothalamic NO, and inflammatory markers	↑ insulin sensitivity, ↓ fasting insulin, ↓ cholesterol, improved cardiometabolic profile	[75,82]
*A. muciniphila* (live)	HFD mice; obesity/NAFLD models	Mucin degradation and repair, mucus-layer thickening, tight-junction enhancement, and bile acid remodeling	↑ mucin turnover and mucus thickness; ↑ FXR–FGF15/19 signaling; ↑ GLP-1; shifts in bile acid pool; ↑ SCFAs (propionate, butyrate)	↓ gut permeability, ↓ endotoxemia, ↓ body weight gain, ↓ hepatic steatosis, ↑ glucose tolerance	[83,84,85,86]

BMI: body mass index, WC: waist circumference, BW: body weight, BF: body fat, T2DM: type 2 diabetes mellitus, FPG: fasting plasma glucose, HbA1c: glycated hemoglobin, HOMA-IR: homeostatic model assessment of insulin resistance, TC: total cholesterol, TG: triglycerides, HDL-C: high-density lipoprotein cholesterol, LDL-C: low-density lipoprotein cholesterol, CRP: C-reactive protein, IL: interleukin, TNF-α: tumor necrosis factor alpha, SCFAs: short-chain fatty acids, GLP-1: glucagon-like peptide-1, CFU: colony-forming units, **↓**: decreased, ↑: increased, LGG primarily supports epithelial barrier integrity and anti-inflammatory signaling, whereas *B. breve* acts primarily through SCFA-mediated metabolic and hormonal effects. *A. muciniphila* is distinguished by mucus-layer repair and bile acid-dependent signaling, and multistrain/synbiotic approaches may offer broader ecosystem-level benefits.

**Table 2:** RCTs of probiotics and synbiotics targeting central adiposity and visceral fat.

Intervention	Population	Duration	Central Adiposity Outcomes	Main Findings	Refs.
*L. gasseri* SBT2055 fermented milk; ~10^6^–10^7^ CFU g^−1^	Adults with abdominal obesity; overweight/obesity	12 weeks	Visceral fat area, BMI, waist, and hip circumferences	Reduced visceral fat area and anthropometric indices; effects evident by week 8 and maintained to week 12	[104]
Multistrain probiotic blend (capsules/fermented product; *Lactobacillus*/*Bifidobacterium* species)	Adults with metabolic syndrome features	8–12 weeks	Body fat mass, BMI, WC, waist-to-height ratio, visceral adipose tissue, liver steatosis grade	Significant reductions in central adiposity and ectopic fat depots versus placebo	[107,114]
*B. breve* + *L. plantarum*	Obese adults	12 weeks	WC, visceral-to-subcutaneous fat ratio	Decreased WC and improved fat redistribution toward less visceral storage	[57,66,68]
Multistrain probiotics (*Lactobacillus*/*Bifidobacterium* combinations)	Children/adolescents with overweight or obesity	8–24 weeks	BMI/BMI z-score, WC	Improved BMI and waist outcomes; some BMI change likely influenced by linear growth	[107]
Synbiotic products (probiotic + prebiotic substrate)	Adults with overweight/obesity; some with BMI class I obesity	12 weeks	WC, body fat %, visceral fat area, adipose distribution	Reduced WC and visceral fat area; stronger effects in men and overweight/class I obesity subgroups in some studies	[95,112]

BMI, body mass index; CFU, colony-forming units. Effects remained significant after adjustment when reported. Some trial effects differed by sex and/or BMI class. In pediatric studies, BMI changes should be interpreted cautiously due to linear growth.

**Table 3:** Pragmatic probiotic and synbiotic recommendations for obesity, T2DM, and NAFLD/T2DM.

Clinical Context	Suggested Formulation	Preferred Matrix	Key Co-Interventions	Strength of Evidence
Overweight/obesity	Multistrain *Lactobacillus*/*Bifidobacterium*; synbiotics if fiber intake is low	Capsule or fermented dairy	Mediterranean-style diet, dietary fiber, and physical activity	Meta-analytic and RCT evidence
T2DM without NAFLD	Multistrain probiotic; synbiotics for enhanced microbiome responsiveness	Capsule, yogurt, or fermented milk	Glycemic diet pattern, dietary fiber, exercise, and background glucose-lowering therapy	Meta-analytic and RCT evidence
T2DM + NAFLD/hepatic phenotype	Synbiotic formulation; consider Akkermansia or postbiotics when appropriate	Synbiotic food or capsule	Mediterranean-style diet, fermentable fiber, weight loss, and physical activity	RCTs and emerging translational evidence
High inflammatory burden/marked dysbiosis	Multistrain probiotic or synbiotic; postbiotics if live strains are less suitable	Capsule or synbiotic food	Energy restriction when indicated, fiber enrichment, and physical activity	Meta-analytic, RCT, and observational evidence
Higher-safety approach	Postbiotics or non-viable probiotic products	Capsule or standardized formulation	Usual dietary and lifestyle measures	Emerging clinical and translational evidence

Safety considerations should be stated clearly in the manuscript. Live probiotics should generally be avoided in severely immunocompromised individuals, critically ill patients, or those with central venous catheters; in these settings, postbiotics or non-viable probiotics may be more appropriate. NAFLD, non-alcoholic fatty liver disease; T2DM, type 2 diabetes mellitus; NGPs, next-generation probiotics.

**Table 4:** Key challenges and proposed solutions in translating probiotic science to metabolic disease care.

Challenge	Consequences	Potential Solutions
Strain specificity	Effects cannot be generalized across species or even within the same species; this contributes to heterogeneous effect sizes and inconsistent trial outcomes.	Conduct strain-resolved RCTs with explicit strain identification, product standardization, and product-specific claims.
Response heterogeneity	Baseline microbiota, diet, host genetics, BMI, and medication use can alter response, limiting generalizability and complicating guideline formation.	Use responder stratification, baseline microbiome profiling, and subgroup analyses to identify likely responders.
Transient engraftment and poor survival	Many strains do not persist after dosing, reducing the durability of the benefit and making effects dependent on continuous use.	Improve delivery with encapsulation, oxygen-tolerance engineering, and synbiotic co-delivery to enhance survival and engraftment.
Small and short RCTs	Most trials are underpowered, short, and use variable endpoints, which limit the detection of clinically meaningful effects and weaken meta-analytic inference.	Run larger, longer, multicenter trials with harmonized metabolic endpoints and adaptive designs.
Limited mechanistic phenotyping	Without microbiome, metabolome, or immune readouts, it is difficult to determine whether SCFAs, bile acids, barrier effects, or inflammation mediate clinical benefits.	Add metagenomics, metabolomics, SCFA biomarkers, bile acid profiling, and immune phenotyping to trial designs.
Safety in high-risk populations	Rare infections have been reported in critically ill, immunocompromised, or catheterized patients, limiting use in vulnerable groups.	Avoid live probiotics in high-risk patients; consider postbiotics or non-viable probiotics where safety is a concern.
Regulatory ambiguity for postbiotics and NGPs	Unclear definitions and inconsistent standards slow clinical translation and complicate product approval and marketing claims.	Establish regulatory standards for strain identification, genomic stability, safety screening, and compositional consistency for postbiotics and next-generation probiotics.
Limited long-term durability data	Benefits may fade after stopping treatment, making long-term clinical impact uncertain.	Use longitudinal follow-up, maintenance-phase designs, and trials testing probiotic-plus-diet strategies.
Unclear interaction with pharmacotherapy	Effects may differ with metformin, SGLT2 inhibitors, or GLP-1 agonists, making interpretation difficult in real-world patients.	Test probiotic strategies in medication-stratified trials and report co-therapies clearly.

NGPs, next-generation probiotics; RCT, randomized controlled trial. Live probiotics should generally be avoided in severely immunocompromised individuals, critically ill patients, or those with central venous catheters; postbiotics or non-viable probiotics may be safer alternatives.

## Data Availability

The original contributions presented in this study are included in the article. Further inquiries can be directed to the corresponding author.

## Abbreviations

The following abbreviations are used in this manuscript:

AhR	Aryl Hydrocarbon Receptor
AUC	Area Under the Curve
BCAAs	Branched-Chain Amino Acids
CFU	Colony-Forming Unit
DXA	Dual-Energy X-ray Absorptiometry
FGF15	Fibroblast Growth Factor 15
FGF19	Fibroblast Growth Factor 19
FIAF	Fasting-Induced Adipocyte Factor
FMT	Fecal Microbiota Transplantation
FXR	Farnesoid X Receptor
GABA	Gamma-Aminobutyric Acid
GLP-1	Glucagon-Like Peptide-1
GPR41	G-Protein Coupled Receptor 41
GPR43	G-Protein Coupled Receptor 43
GPR109A	G-Protein Coupled Receptor 109A
HDAC	Histone Deacetylase
HFD	High-Fat Diet
HOMA-IR	Homeostatic Model Assessment of Insulin Resistance
IL-10	Interleukin-10
IL-6	Interleukin-6
LPS	Lipopolysaccharide
LPL	Lipoprotein Lipase
NAFLD	Non-Alcoholic Fatty Liver Disease
NF-κB	Nuclear Factor Kappa B
NGP	Next-Generation Probiotics
NOD	Non-Obese Diabetic
PYY	Peptide YY
RCT	Randomized Controlled Trial
SCFAs	Short-Chain Fatty Acids
SIRT1	Sirtuin-1
SOD	Superoxide Dismutase
SREBP1c	Sterol Regulatory Element-Binding Protein 1c
T2DM	Type 2 Diabetes Mellitus
TEER	Transepithelial Electrical Resistance
TGF-β	Transforming Growth Factor Beta
TGR5	Takeda G-Protein Receptor 5
TLR	Toll-Like Receptor
TNF-α	Tumor Necrosis Factor Alpha
Treg	Regulatory T Cell
ZO-1	Zonula Occludens-1
